# Band offset engineering at C_2_N/MSe_2_ (M = Mo, W) interfaces†

**DOI:** 10.1039/d2ra00847e

**Published:** 2022-04-20

**Authors:** Amine Slassi

**Affiliations:** Istituto Nanoscienze-CNR Via Campi 213a I-41125 Modena Italy a.slassi22@gmail.con amine.slassi@nano.cnr.it; Laboratory for Chemistry of Novel Materials, Université de Mons Place du Parc 20 7000 Mons Belgium

## Abstract

Stacking layered two-dimensional materials in a type-II band alignment block has provided a high-performance method in photocatalytic water-splitting technology. The key parameters in such heterostructure configurations are the valence and conduction band offsets at the interface, which determine the device performance. Here, based on density functional theory calculations, the bandgap and band offsets at C_2_N/MSe_2_ (M = Mo, W) interfaces have been engineered. The main findings demonstrate that the C_2_N monolayer interacts with both MoSe_2_ and WSe_2_ monolayers through weak van der Waals interactions. These heterostructures possess a narrower indirect bandgap and a typical type-II heterostructure feature, being suitable for promoting the separation of photogenerated electron–hole pairs. The calculated Gibbs free energy of hydrogen adsorption demonstrates a reduction in the overpotential, towards the hydrogen evolution reaction, upon forming heterostructures. To further tune the bandgap values and band offsets of heterostructures, the external perturbations are included through a vertical strain and finite electric field. It is found that both the vertical strain and electric field strongly modulate the bandgap values and the magnitude of the band offsets, while the typical type-II band alignment remains preserved. It is noticeable that the band offset magnitudes of the C_2_N/MoSe_2_ and C_2_N/WSe_2_ heterostructures are more sensitive to an external electric field than to a vertical interlayer strain.

## Abstract 2

Stacking layered two-dimensional materials in a type-II band alignment block has provided a high-performance method in photocatalytic water-splitting technology.

## Graphical abstract

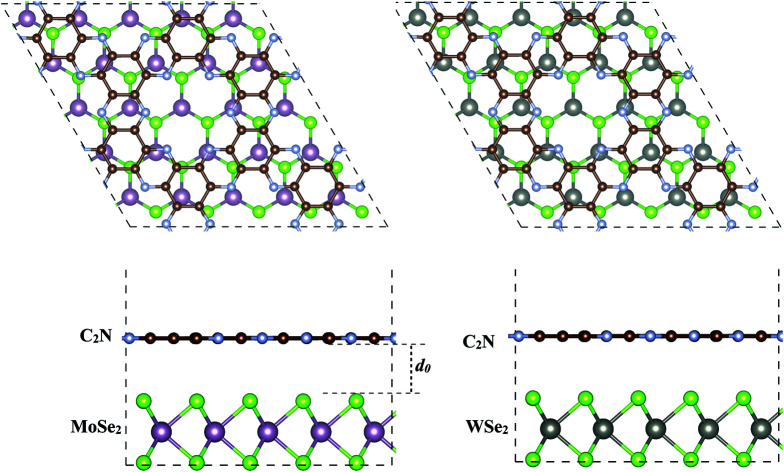

## 1 Introduction

Porous carbon nitride (C_2_N) has attracted tremendous interest as a metal-free two-dimensional (2D) semiconductor photocatalyst for hydrogen production from visible-light driven water splitting.^1,2^ This interest stems from its fascinating features such as an appropriate direct band gap value of 1.96 eV covering a considerable part of the visible-light absorption,^3^ high structural stability^4^ and suitable band edge positions.^5^ Several experimental studies demonstrated that 2D C_2_N can be an efficient photocatalyst in the hydrogen evolution reaction.^1,2^ However, similar to the g-C_3_N_4_ material, the fast recombination of photoinduced electron–hole pairs also prevents achieving a high photocatalyst performance of the C_2_N material.^3^

A new paradigm in photocatalytic devices is to vertically stack 2D materials, with different ionization potentials and electronic affinities to generate a donor–acceptor heterostructure photocatalyst, which has demonstrated to be the most effective approaches for suppressing the electron–hole pairs recombination and improving the photocatalytic performance.^2,6,7^ Recently, several C_2_N-based vdW heterostructures such as C_2_N/MoS_2_, C_2_N/Janus monochalcogenides,^8^ C_2_N/aza-CMP,^9^ C_2_N/h-BN,^10^ C_2_N/cobalt-oxide,^2^ C_2_N/CdS,^11^ C_2_N/g-C_3_N_4_ (ref. 12) and C_2_N/WS_2_ (ref. 13) were studied. Unsurprisingly, the results demonstrated that a significant enhancement in the photocatalytic efficiency can be achieved in such vdW heterostructures as compared to the single-layered form. For instance, Mahmood *et al.*^2^ experimentally demonstrated that the stacking C_2_N with cobalt-oxide, in a heterostructure stack, results in higher catalytic activities for hydrogen (H_2_) production with a generation-rate comparable to that of the best reported values for catalysts containing precious noble metals.

Whatever, the band edge alignment of constituting components, with respect to each other, is a key parameter determining the heterostructure device performance.^14,15^ For photocatalytic applications, a heterostructure with a type-II band alignment configuration demonstrated to efficiency separate the photogenerated electron–hole pairs at the interface for highly efficient water reduction and oxidation.^6,16^ In such a type-II configuration, the magnitude of the conduction band offset (CBO; defined as the difference in the electron affinities of two constituting components) and valence band offset (VBO; the difference in the ionization potentials) determine the magnitude of the built-in electric at the interface that in turn separates the photogenerated electrons and holes in space. Koda *et al.* demonstrated, by the first principles calculations, that tailoring the band offsets (CBO and VBO) at phosphorene/TMDs strongly modulate the electronic properties of heterostructures.^17^ Zhang *et al.* showed that the band alignment engineering is a good approach to achieve a higher photocatalyst performance of MoS_2_/GaN heterostructure for hydrogen generation.^18^

In the present study, the electronic properties of C_2_N/MoSe_2_ and C_2_N/WSe_2_ heterostructures have been studied by employing the density functional theory (DFT). The band energy diagrams show a band alignment type II with large band offsets in the valence band maximums and conduction band minimums, which providing strong driving forces to pump the photo-generated electrons from MoSe_2_ (WSe_2_) layer to C_2_N layer, and the photo-generated holes in the opposite directions. The band offsets and band gap values have been further engineered by modulating the interlayer electronic interactions and applying external perturbations such as a interlayer strain and a finite electrical field.

## 2 Calculation methods

All calculations were performed by using the Vienna ab initio simulation package (VASP). The interaction ion-cores and valence-electrons were described by projected – augmented wave (PAW) method with a cutoff energy of 600 eV.^19,20^ GGA within Perdew, Burke, and Ernzerhof (PBE) approach was used for the exchange correlation functional.^21^ Due to the presence of the van der Waals (vdW) interactions in our studied heterostructures, the Grimme 2D was included.^22^ Monkhorst–Pack 2 × 2 × 1 and 5 × 5 × 1 *k*-point grids are used to sample the Brillouin zone (BZ) for geometry optimizations and the electronic structure calculations, respectively. All ions are allowed to be relaxed till their residual Hellmann–Feynman forces are less than 0.01 eV Å^−1^.

The optical absorptions of C_2_N monolayer as well two MoSe_2_/C_2_N and WSe_2_/C_2_N heterostructures were calculated by the following formula:^23^1
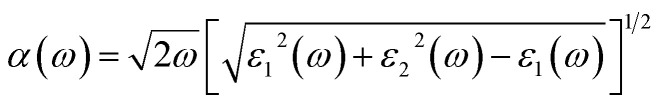
where, *ε*_1_ and *ε*_2_ represent the real and imaginary parts of the dielectric function based on the Drude–Lorentz model.^24^ A detailed description of optical equations was previously reported in literature.^23,25–27^

## 3 Results and discussion

### 3.1 . Properties of heterostructures

**Fig. 1:**
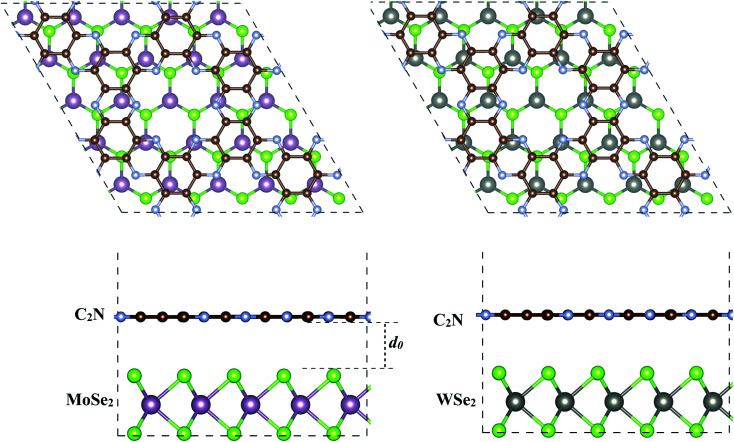
Top and side views of the optimized of (a) C_2_N/MoSe_2_ and (b) C2N/WSe_2_ heterostructures. *d*_0_ denotes the equilibrium interlayer distance.

The geometry relaxation of isolated C_2_N, MoSe_2_ and WSe_2_ monolayers was first considered. The lattice parameters for C_2_N, MoSe_2_ and WSe_2_ monolayers are calculated to be 3.32, 3.31 and 8.318 Å, respectively, which are in good agreement with the experimental and previous theoretical results.^17,28,29^ To construct the heterostructures, 5 × 5 × 1 unit cells of MoSe_2_ (WSe_2_) were placed on 2 × 2 × 1 unit cells of C_2_N resulting in a low lattice mismatch, less than 0.5% for both heterostructures, which implies that the effect of lattice mismatch on the electronic properties of individual monolayers remains unaffected in this study. The supercell heterostructures contain 147 atoms, including 72 C, 50 Se, and 25 Mo (W) atoms. The equilibrium geometries of MoSe_2_/C_2_N and MoSe_2_/C_2_N heterostructures are shown in Fig. 1.

Upon heterostructure configurations, the C_2_N, MoSe_2_ and WSe_2_ monolayers keep their plane and hexagonal atomic network intact without any remarkable distortions. The calculated equilibrium interlayer distance between C_2_N and MoSe_2_ (WSe_2_) is 3.36 Å (3.32), close to the interlayer distance of 3.34 Å at C_2_N/MoS_2_ (ref. 7) and 3.33 Å WS_2_/C_2_N heterostructures.^13^ The corrugation of C_2_N is still less than 0.043 Å (0.065) in MoSe_2_/C_2_N (WSe_2_/C_2_N) heterostructure, which is smaller than the significant buckled height at C_2_N/g-C_3_N_4_ heterostructure.^12^

To quantitatively characterize the interlayer interactions, the binding energy (*E*_b_) is estimated by following equation:2
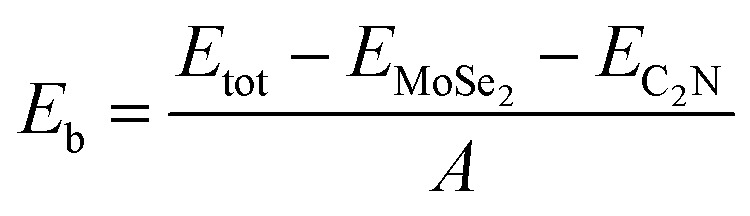
where *E*_tot_, *E*_MSe2_ and *E*_C2N_ are energies of heterostructure, isolated MSe_2_ (M = Mo or W) monolayer and isolated C_2_N monolayer, respectively. *A* is the surface area at interface. At the equilibrium interlayer distances, the calculated binging energies are −0.23 and −0.27 J m^−2^ for C_2_N/MoSe_2_ and C_2_N/WSe_2_, respectively. These values are close to the calculated binding energy values reported for TMD based van der Waals heterostructures.^30–33^ This also indicates that the formation of C_2_N and MoSe_2_ (WSe_2_) heterostructure is exothermic and considered as a vdW heterostructure, which is similar to what reported for MoS_2_/C_2_N and WS_2_/C_2_N heterostructures.^7,13^

**Fig. 2:**
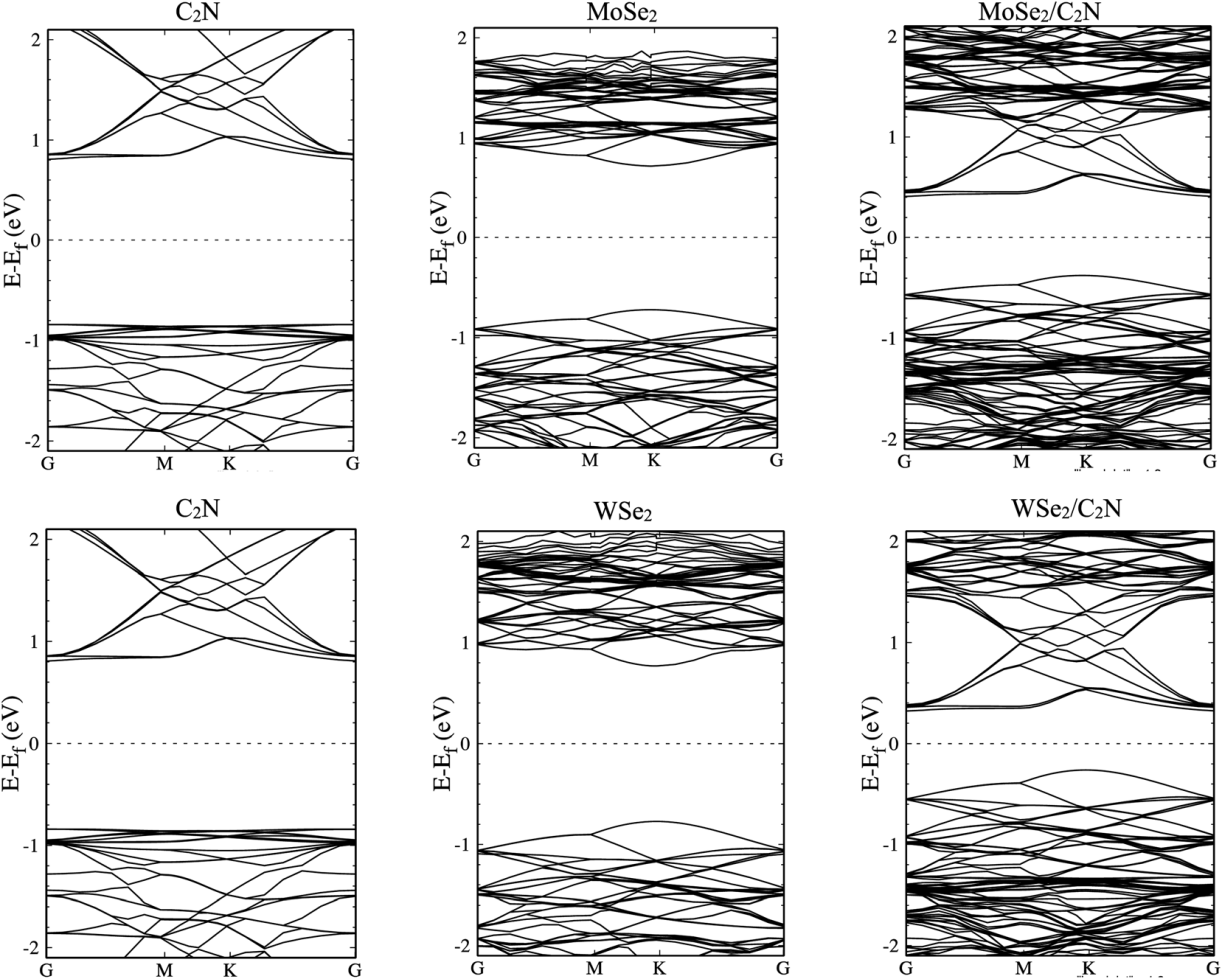
Band structures of isolated monolayers and their heterostructures.

The electronic structures of MoSe_2_/C_2_N and WSe_2_/C_2_N heterostructures at equilibrium interlayer distance were analyzed. The obtained band structures are shown in Fig. 2, indicating that both C_2_N/MoSe_2_ and C_2_N/WSe_2_ heterostructures are semiconductors with indirect band gaps at *G*–*K* points and values of 0.8 eV and 0.58 eV, respectively, smaller than the direct band gaps of individual layers of 1.73 eV (C_2_N) at *G*-point, 1.56 eV (MoSe_2_) and 1.67 eV (WSe_2_) at *k*-point, due mainly to the band offsets at the band edges. Thus, the electrons can be easier photo-excited from the valence band (VB) to the conduction band (CB) in heterostructures under the IR and visible light radiations, which improves the electron–hole photo-generation rate as compared to the individual monolayers. Note that the bandgap values calculated at the PBE level are lower than the quasi-particle bandgap values found at the GW many-body theory level^34^ owing to the shortening of GGA in accurately describing the exchange–correlation (xc) functional. Notwithstanding, comparing at the GGA level the relative values of the bandgap of two different materials and the relative alignment of their valence or conduction band edges are still reasonable, which will not qualitatively affect the interpretation of band values based on my calculations.^6^ Indeed, the use of GW induces only a rigid band shift of VBM and CBM by the same amount but in the inverse directions with respect to the same band-gap-center by DFT-calculations.^14,35^

The calculated density of states, as shown in Fig. S1 (ESI†), show that the top part of the valence band is formed by states from MoSe_2_ (WSe_2_) and the bottom part of the conduction band is formed by states from C_2_N for both heterostructures. The deep orbital analysis shows that the valence band maximum (VBM) is dominated by Mo-d_*x*^2^−*y*^2^_ orbital (W-d_*x*^2^−*y*^2^_), while the conduction band minimum (CBM) is dominated by N-p_*z*_ orbital contributions. On the other hand, no overlap of MoSe_2_ (WSe_2_) orbitals with those from C_2_N layer is observed at band edges.

**Fig. 3:**
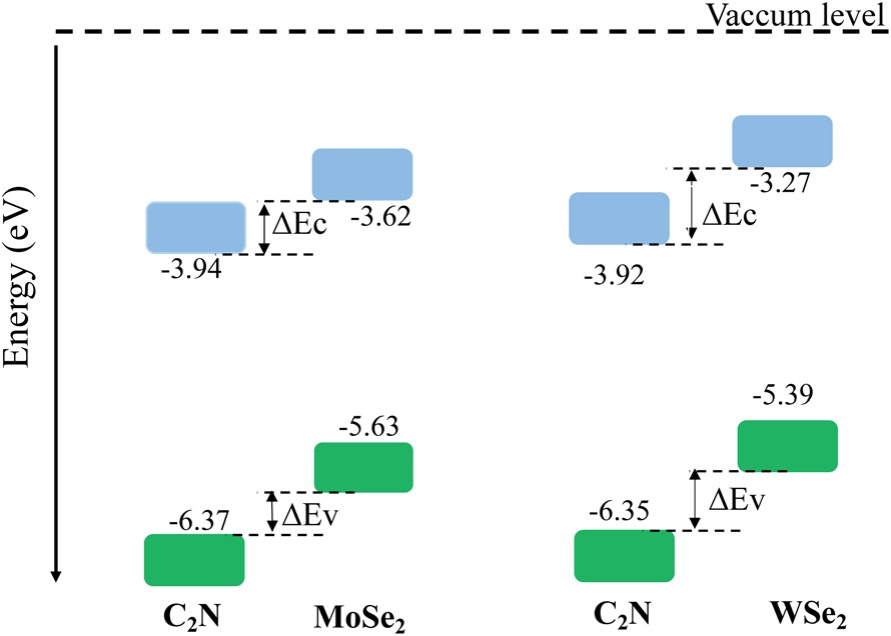
Band alignments of heterostructures at interactive configurations.

The calculated electronic band alignment diagrams of MoSe_2_/C_2_N and WSe_2_/C_2_N heterostructures, at equilibrium phases, are shown in Fig. 3, in which the zero is referenced to the vacuum level. It is found that the CBM and VBM of the C_2_N layer are lower in energy than those of MoSe_2_ (WSe_2_) layer, which indicates that the MoSe_2_/C_2_N heterostructure (WSe_2_/C_2_N) is expected to form a type II band alignment configuration. This would lead to a significant enhancement in the photocatalytic H_2_ generation.^6^ The calculated VBO and CBO between C_2_N and MoSe_2_ (WSe_2_) layers are Δ*E*_v_ = 0.84 eV (1.05) and Δ*E*_c_ = 0.65 eV (0.96) respectively. These values are larger than the CBO (0.54 eV) and VBO (0.67 eV) at WS_2_/C_2_N heterostructure.^13^ For the sake of comparison, I calculated the band alignment of heterostructures at HSE06 level, see Fig. S2.† Overall, the band alignment at HSE06 level shows similar type-II band alignment configuration, while the difference being only a small variation in the band offsets (CBO and VBO). This implies that although the GGA-PBE significantly underestimates the bandgap values of individual components, the main interpretation would not be qualitatively affected when one considers the engineering of the band offsets. To avoid the high computational cost at HSE06 level, I therefore focused on GGA-PBE for engineering the band offsets at heterostructures under different external perturbations.

On the other hand, these large band offsets would establish a strong built-in electrical field breaking the photo-generated electron–hole exciton bonds at the interface, which then drives the generated electrons and holes to the opposite special sides across the interface. Hence, under the light illumination, the electrons will be excited form the valence bands of both C_2_N and MoSe_2_ (WSe_2_) to their conduction bands, therefore, the photo-generated electrons in the conduction band of MoSe_2_ (WSe_2_) can be spontaneously transferred to the conduction band of C_2_N due to the generated strong electrical force. Meantime, the photo-generated holes in the valence band of C_2_N can be also spontaneously transferred to the valence band of MoSe_2_ (WSe_2_). This makes C_2_N a negatively charged layer and MoSe_2_ (WSe_2_) a positively charged layer, forming a n–p heterojunction. Therefore, such an interfacial charge separation enhances the electron–hole photo-generations by suppressing their recombination.

Sequentially, I estimated the charge transferred between C_2_N and MoSe_2_ (WSe_2_) layer by using the Bader charge analysis. The amount of electron transferred from MoSe_2_ (WSe_2_) to C_2_N is estimated to be 0.18 |e| (0.20 |e|) per supercell. These results are in good trend with what predicted from the band alignment diagrams. Moreover, the electron transfer across interface leads to a formation of an interfacial dipole layer and an accompanying potential step Δ*V* due to bending band as shown in Fig. S3 (ESI†). The Δ*V* step is defined as the difference between the right and left values of the potential profile with respect of the interface, which is considered as a barrier height of the carrier injections in heterojunction (see Fig. S3 in ESI†). Indeed, the formation of bending band in the heterostructures is suitable for charge separation. The calculated values of Δ*V* are 0.13 eV and 0.12 eV for C_2_N/MoSe_2_ and C_2_N/WSe_2_, respectively.

**Fig. 4:**
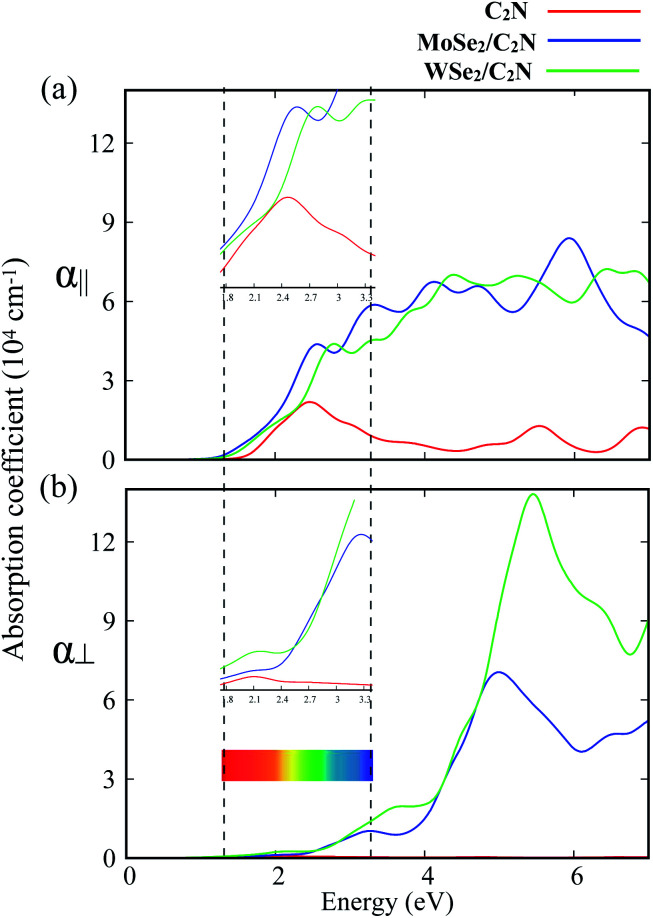
Calculated absorption spectra for C_2_N, MoSe_2_/C_2_N and WSe_2_/C_2_N along: (a) parallel (*α*_∥_) and (b) normal (*α*_⊥_) direction of the C_2_N monolayer plane, respectively. The dashed lines denote the visible light range.

To further explore the combined effects of C_2_N and MoSe_2_ (WSe_2_), I investigated the optical properties of the isolated C_2_N monolayer as well as the MoSe_2_/C_2_N and WSe_2_/C_2_N heterostructures. The absorption spectra along two main polarization vectors, parallel (*α*_∥_) and normal (*α*_⊥_) to the C_2_N plane are shown in Fig. 4. From the first view, the optical absorption strength of isolated C_2_N along parallel polarization (*α*_∥_) is higher than that along the normal (*α*_⊥_) polarization vector within the IR and visible light ranges. The absorption threshold for isolated C_2_N monolayer is lied at the photon energy of 1.67 eV, which is close to bandgap energy. This is due mainly to electron transitions from VBM to CBM; while the other peaks lying above this threshold are mainly originated from the different inter-band electron transitions from VB to CB of isolated C_2_N monolayer.

Upon heterostructure configurations, the optical absorption strength over the visible light range is significantly enhanced for both MoSe_2_/C_2_N and WSe_2_/C_2_N heterostructures with a red-shift in the absorption threshold towards low energy photons, which is an extra value for C_2_N based photocatalyst. This red shift of the absorption edge is mainly due to effect of interfacial interactions and reduced band gap values upon heterostructure configuration, the photogenerated electrons would be directly excited from the VBM of MoSe_2_ (WSe_2_) to the CBM of C_2_N component. For the sake of comparison, the enhancement of optical absorption is obviously higher at WSe_2_/C_2_N than that at MoSe_2_/C_2_N heterostructure; this is rationalized by the fact that the magnitude of band offsets at WSe_2_/C_2_N is larger.

### 3.2. Hydrogen evolution reaction

**Fig. 5:**
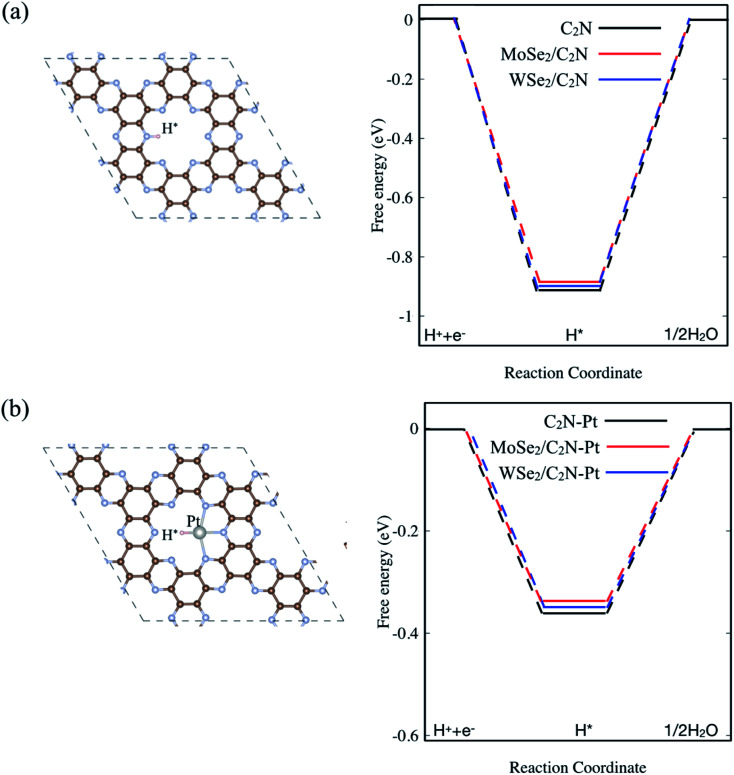
Calculated Δ*G*_H*_ diagram for: (a) pristine and (b) Pt anchored on isolated C_2_N surface. The top view of H adsorbed on pristine and Pt anchored on C_2_N are inserted on (a) and (b), respectively.

In order to assess the effect of the interface formation on the hydrogen evolution reaction (HER) efficiency, the Gibbs free energy diagram of hydrogen adsorption (Δ*G*_H*_) was quantified. More detailed information is found in the ESI.† Previous studies demonstrated that the Gibbs free energy of hydrogen adsorption (Δ*G*_H_) is the reasonable descriptor of HER activity.^36^ Of note, the value of (Δ*G*_H*_) close to zero results in a fast hydrogen release step and therefore suitable material for HER. The lower value of (Δ*G*_H*_) indicated strong bonds with H atoms, resulting in a slow hydrogen evolution process. For the sake of completeness, I also considered a single Pt atom anchored on the C_2_N surface. Here, the energetic calculation tests of Pt-adsorption over different sites indicated that Pt atom prefers to be anchored on the cavity site of C_2_N forming bonds with N atoms, which is similar to previous calculations.^37^ On the other hand, the hydrogen atom in the pristine monolayer/heterostructure prefers to chemically bind on top of N sites, indicating that the N atom sites are the active sites for HER reaction. For the Pt-anchored on C_2_N, the Pt atom sites are found to be active sites. Thus, the diagram of free energy for the hydrogen adsorption (Δ*G*_H*_) is computed as shown in Fig. 5. The value of Δ*G*_H*_ for the isolated C_2_N sheet is calculated to be −0.91 eV, which indicates a strong chemical bond between H and N atoms. This would require an overpotential of +0.91 eV for an overall H_2_ evolution. Upon heterostructure configuration, the value of (Δ*G*_H*_) decreases by 18 and 11 meV for MoSe_2_/C_2_N and WSe_2_/C_2_N, respectively. This is rationalized by the fact that the charge transfer from MoSe_2_ (WSe_2_) sheet to C_2_N modifies the charge density over the active sites, which in turn balance the photocatalytic activity towards the HER. Thus, the controlling of the band alignment and charge transfer across the interface would optimize the catalytic efficiency of C_2_N-based heterostructures. It should be interesting to note that the anchoring of a single Pt atom on C_2_N surface significantly decreases the overpotential toward HER, while the values of (Δ*G*_H*_) further decrease upon heterostructure configurations.

### 3.3. Applying a vertical strain

**Fig. 6:**
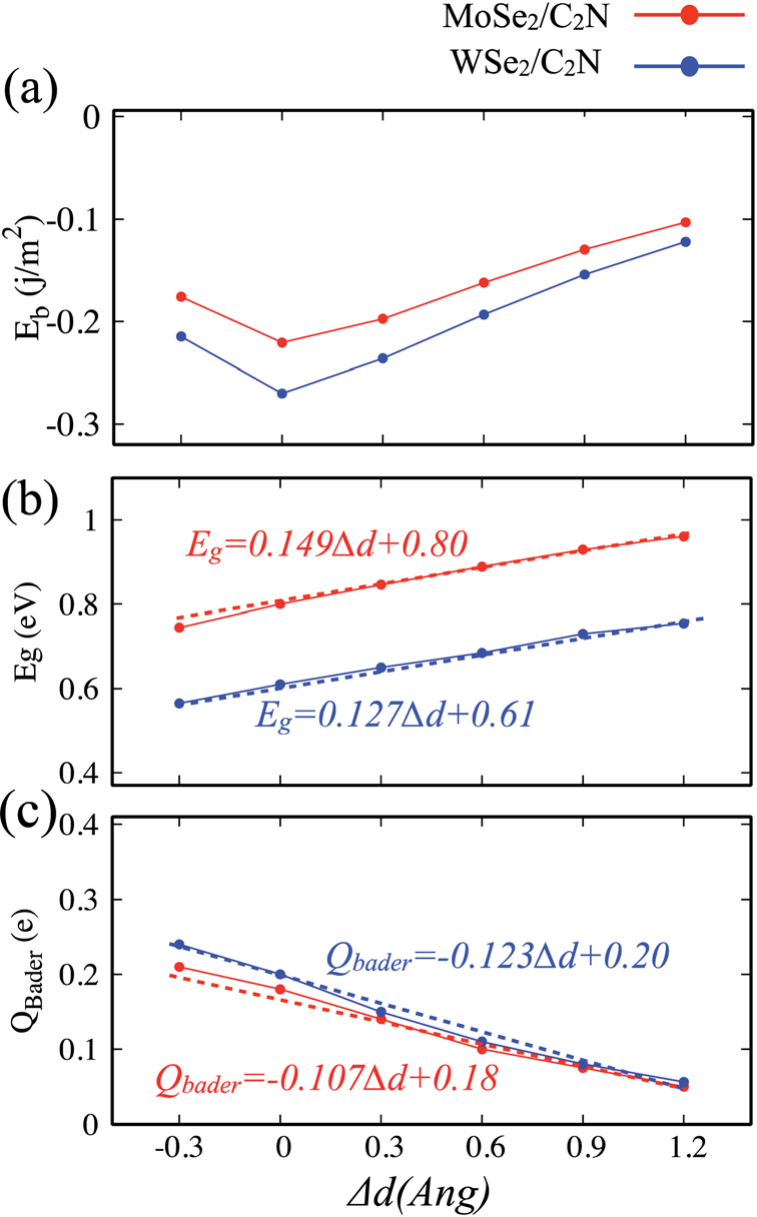
Variation as a function of interlayer distance of: (a) binding energy, (b) bandgap energy and (c) Bader charge transfer. Red curve for C_2_N/MoSe_2_ and black curve for C_2_N/WSe_2_ heterostructures. Zero of the interlayer distance scale is put at the equilibrium position.

The electronic properties of 2D heterostructures can be modulated by artificially varying the interlayer distance. By varying the vdW interlayer spacing in the heterostructures, it is possible to change the binding energy, bandgap values and charge transfer between the constituents of the hetero-structure due to the alteration in the interlayer electronic coupling. Such a vertical strain has been proven to be an effective approach to alter the electronic structures of heterostructures in many theoretical studies.^38–40^ In the experimental point of view, the applied vertical strain to vdW hetero-structures can be applicable by employing the diamond anvil cells.^41^ In our calculations, the strain was applied by varying the interlayer distance by −0.3, 0.3, 0.6, 0.9 and 1.2 Å with respect of equilibrium distance (*d*_0_). The evolutions of binding energy, bandgap and Bader charge transfer values at heterostructures as a function of the applied vertical strain are shown in Fig. 6.

Here, when both tensile and compressive strain are applied, the binding energy is slightly decreased, as shown in Fig. 6-a, which implies that the thermodynamic stability is slightly perturbated by such a moderate applied vertical strain.

The evolution of the band gap value as a function of the applied strain is shown in Fig. 6-b. The bandgap is still indirect regardless the tensile/compressive applied strain. Upon applying a compressive vertical strain, the band gap decreases due mainly to enhancement in the interaction between the orbital p_*z*_ of C_2_N layer and orbital d_*x*_^2^_−*y*_^2^ of MoSe_2_ (WSe_2_) layer. Therefore, the edge absorption would be red-shifted resulting in an enhanced optical absorption over the IR and visible regions. In contrast, applying a tensile vertical strain leads to an increase in the bandgap value which must be due to suppressing of interaction between C_2_N monolayer and MoSe_2_ (WSe_2_) monolayer. In this last case, the absorption edge would be blue-shifted.

The modification in the interaction between C_2_N and MoSe_2_ (WSe_2_) is also associated to the charge transfer across the interface. The Bader charge transferred from the MoSe_2_ (WSe_2_) layer to C_2_N layer as a function of the vertical strain is also calculated, as shown in Fig. 5-c. At equilibrium interlayer distance, the charge transferred is calculated to be 0.18 |e| (0.20 |e|). Upon the interlayer distance decreases, the charge transfer considerably increases. Whereas, the charge transfer decreases as the interlayer increases. This demonstrates a strong dependence of the charge transfer on the vertical strain and significant modulation of interlayer electronic process and the band alignment at interface.

**Fig. 7:**
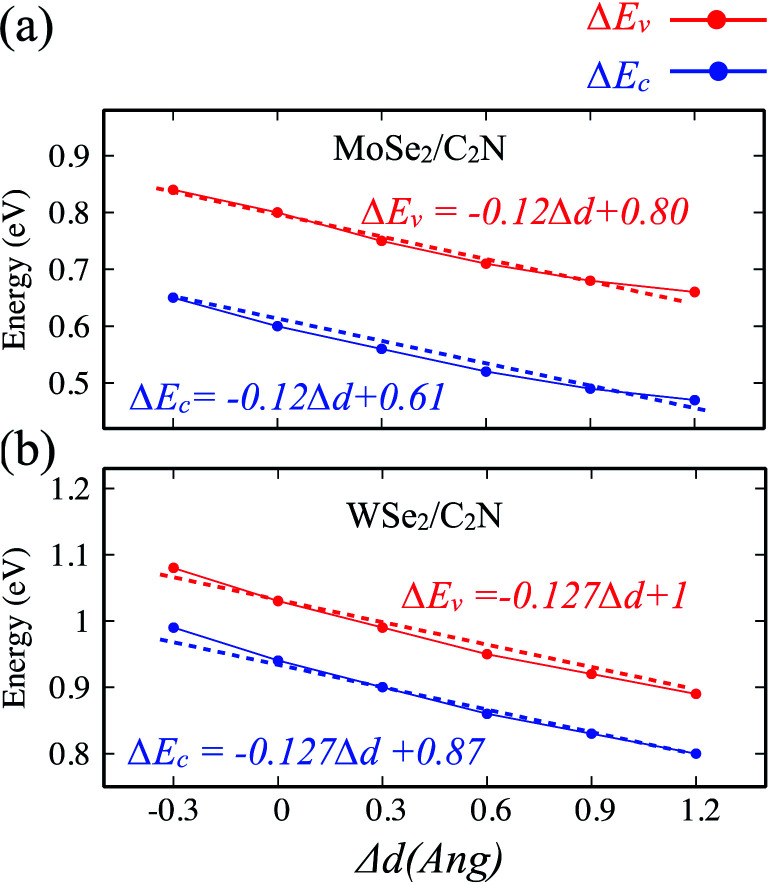
Variation of band offsets Δ*E*_c_/Δ*E*_v_ as a function of the vertical strain for (a) MoSe_2_/C_2_N and (b) WSe_2_/C_2_N heterostructures.

The effect of the vertical strain on the band offset is shown in Fig. 7. Interestingly, the band alignment type II is still preserved upon applying a tensile/compressive strain. The increasing in the interlayer distance results in a decrease of both VBO and CBO of heterostructures due to weakening in the interlayer interaction.

By placing the layers very far each other, the VBO and CBO trend towards those of isolated systems. In contrast, the decreasing in the interlayer distance leads to increasing in VBO and CBO due to increasing in the interlayer electronic interaction and orbital overlapping.

### 3.4. Applying an external electric field

**Fig. 8:**
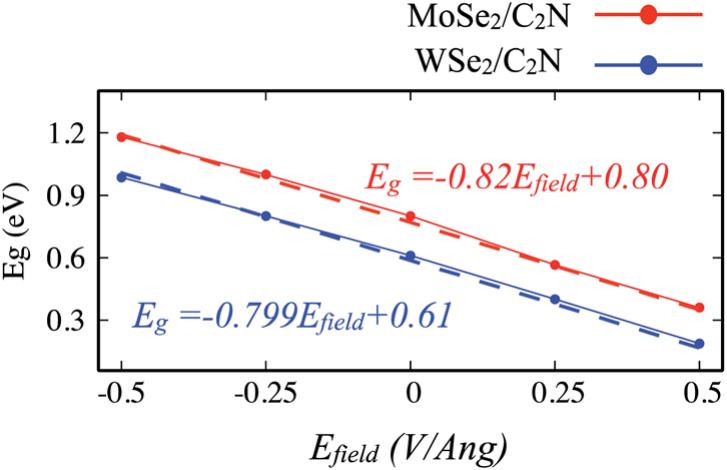
Variation of band gap values of heterostructure under applied vertical electrical field *E*_field_.

Another strategy to tune the interlayer interactions at heterostructure interfaces is to apply a vertical external electric field in the normal direction. Although the band energy diagram, work function, band gap, and charge transfer in heterostructure are strongly depend on the magnitude of the external electric field and its direction, it is found to have a negligible effect on the interlayer distance between MoSe_2_ (WSe_2_) layer and C_2_N layer. Here, I evaluated the effect of a finite external electric field varying from −0.5 to 0.5 V Å^−1^ by a step of 0.25 V Å^−1^ on the band offsets and the band gap values of heterostructures. Such external electric field can be also interpreted as a bias for fermi. For individual C_2_N and MoSe_2_ (WSe_2_) monolayers, their band gaps are almost unaffected by the external electric field. Fig. 8 shows the change in the band gap value of MoSe_2_/C_2_N (WSe_2_/C_2_N) under the external electric field.

The positive direction of the electric field means that the applied field is pointing from MoSe_2_ (WSe_2_) layer towards C_2_N layer. At a positive electric field from 0.0 to 0.5 V Å^−1^, the band gap decreases from 0.8 eV (0.62 eV) to 0.53 eV (0.34 eV) for MoSe_2_/C_2_N (WSe_2_/C_2_N). While, at the negative electric field from −0.5 to 0.0 V Å^−1^, the band gap increases. This change in the band gap values is mainly due to the modification in the band alignment in presence of an electrical field, which implies a high sensitivity of the features of the heterostructure to an external electric field.

**Fig. 9:**
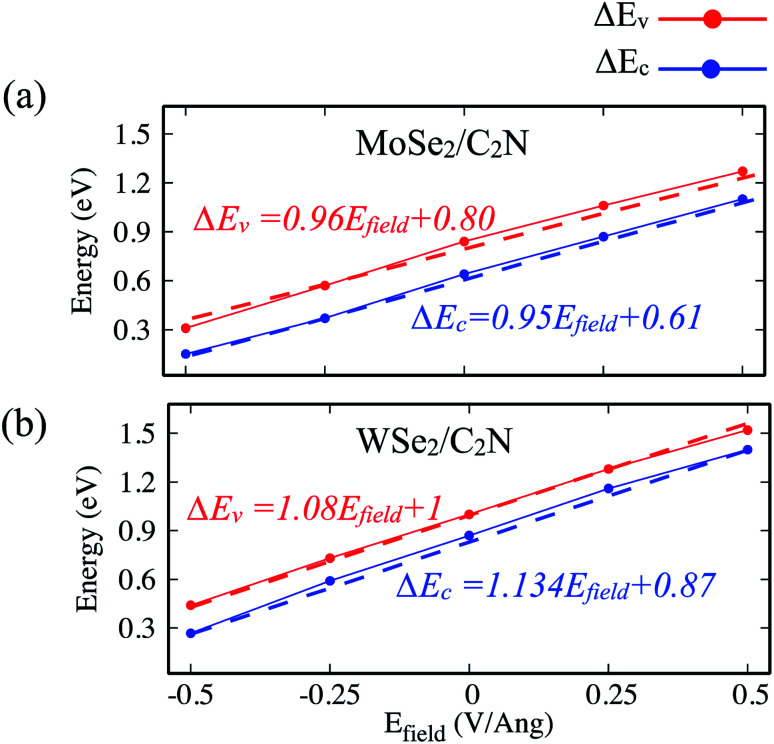
Variation in the Δ*E*_c_/Δ*E*_v_ as a function of vertical electric field (*E*_field_).

Indeed, it has been already proved from the previous theoretical calculations and experimental measurements that the external electric field can alter the band alignment of heterostructures.^38,42^ The variation in the band offsets at interface under an external electric field is shown in Fig. 9. When a positive electric field is applied, the VBM and CBM of MoSe_2_ (WSe_2_) layer trend to shift up with respect of the equilibrium fermi while the VBM and CBM of C_2_N layer shifts down (as shown in Fig. S4†), leading to an increase in VBO and CBO as demonstrated in Fig. 9. Similar trend is also observed at hetero-bilayers of TDMCs.^43^ In the case of a negative electrical field, the VBM (CBM) of MoS_2_ and C_2_N trend to shift toward each other leading to decreasing in VBO and CBO magnitude. More important, the type-II band alignment at C_2_N/MoSe_2_ and C_2_N/WSe_2_ heterostructures is also preserved upon applying a finite external electric field *E*_field_ range from −0.5 V Å^−1^ to 0.5 V Å^−1^.

## 4 Conclusion

In summary, I have studied the thermodynamic stability and electronic structures of C_2_N/MoSe_2_ and C_2_N/WSe_2_ heterostructures under the vertical strain and finite external electric field by using the DFT method. The interlayer distance and binding energy have demonstrated that the C_2_N monolayer holds with MoSe_2_ or WSe_2_ monolayer through the weak vdW interactions. This indicates that the formation of these heterostructures is exothermic. The band structures have shown that the heterostructures exhibit a narrower indirect band gap as compared to individual components, which boost the optical absorption over the visible and IR lights. The band alignment diagram has indicated the stabilization of a type-II band alignment configuration at heterostructures with large band offsets, implying a good performance of charge separation of photogenerated electron–hole pairs. The calculated Gibbs free energy of hydrogen adsorption have demonstrated a reduction in the overpotential, towards hydrogen evolution reaction, upon forming heterostructures, which is rationalized by charge transfer across interface. This indicates that the control of band offsets at interface can optimize the HER efficiency. The application of external perturbations includes the vertical strain and finite electrical field can modulate the band alignments. By applying a compressive strain, the band gap values have decreased and the band offsets have increased. Moreover, a similar trend has been observed upon applying a positive finite external electric field pointing from MoSe_2_ (WSe_2_) layer towards C_2_N layer. The magnitude of decrease/increase in the band gap energy/band offsets at MoSe_2_/C_2_N and WSe_2_/C_2_N heterostructures is bigger upon applying a finite electrical field than applying a vertical strain. The results have demonstrated that the type-II configuration at MoSe_2_/C_2_N and WSe_2_/C_2_N heterostructures is useful for photocatalytic water splitting towards hydrogen production. The applying either a vertical strain or a finite electrical field can bring an additional enhancement without switching the type-II band alignment configuration. The decrease in the band gap leads to a red shift in the absorption spectrum and allows to further harvest the IR and visible light as compared to individual components. The increase in the band offsets leads to strengthen the built-in electrical field across the interface; therefore, the separation of photo-generated electron–hole pairs would be more effective.

## Funding

This work was funded by EC through H2020-DT-NMBP-11-2020 project GA no. 953167 (OpenModel).

## Conflicts of interest

There are no conflicts to declare.

## Supplementary Material

RA-012-D2RA00847E-s001
