# Supplementary material for: Band offset engineering at C2N/MSe2 (M = Mo, W) interfaces
Source: RSC Adv. 2022 Apr 20;12(19):12068–77. doi: 10.1039/d2ra00847e (PMC9019558; doi:10.1039/d2ra00847e)
Supplement: RA-012-D2RA00847E-s001 [file RA-012-D2RA00847E-s001.pdf]

Electronic Supplementary Material (ESI) for RSC Advances.
This journal is © The Royal Society of Chemistry 2022

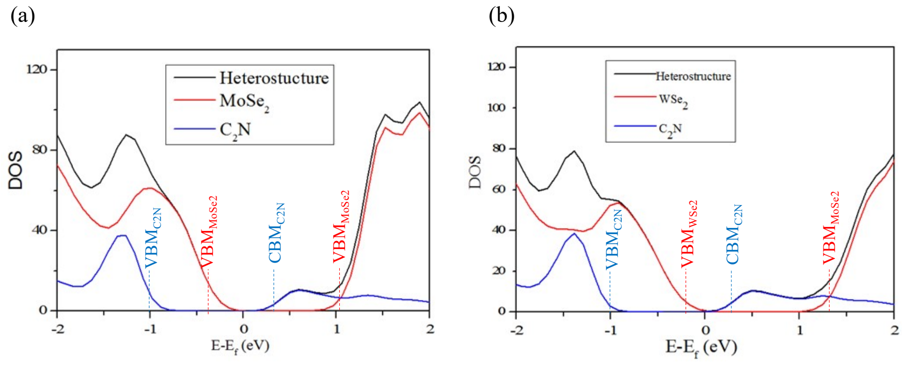

***Figure S1**: Project density of states of (a) $C_2N/MoSe_2$ and (b) C_2N/WSe_2 heterostructures, respectively, at equilibrium geometry.*

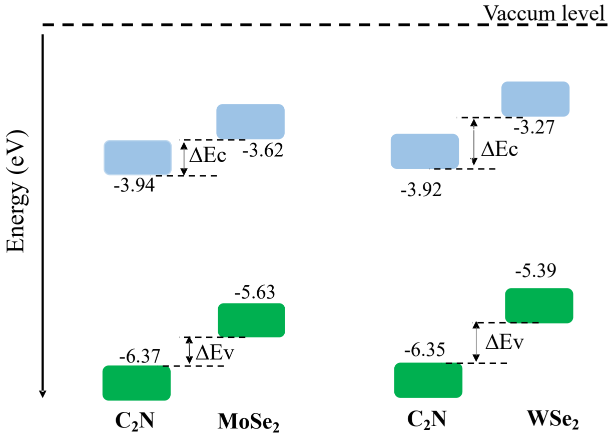

***Figure S2** : Band alignments of heterostructures at interactive configurations at HSE06 level.*

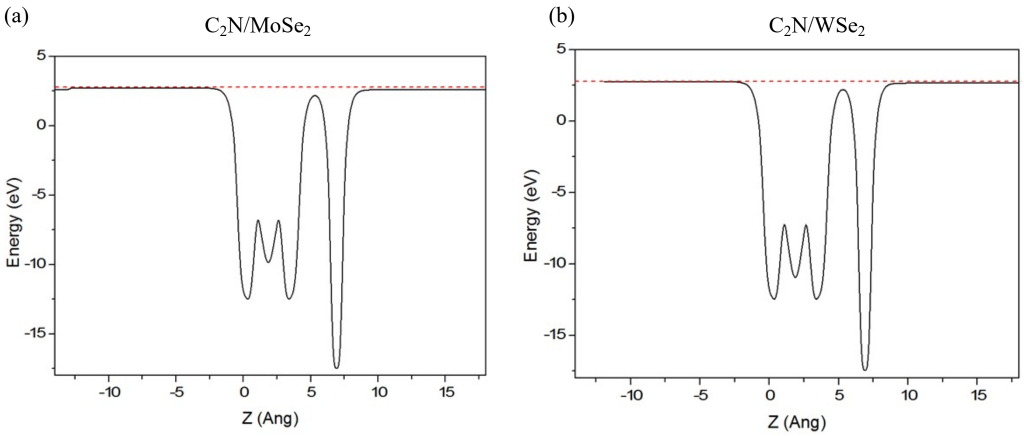

Figure S3: *Figure S1: Plane-averaged electrostatic potential along the normal direction of (a) $C_2N/MoSe_2$ and (b) C_2N/WSe_2 heterostructures, respectively, at equilibrium geometry.*

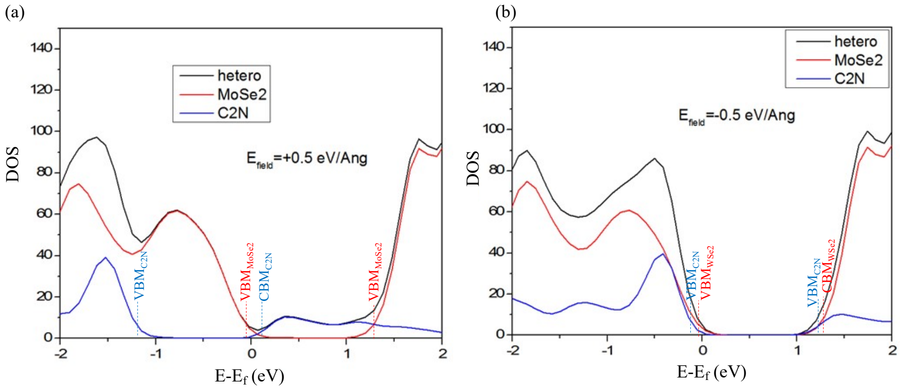

Figure S4: *Figure S1: Representative Project density of states of $C_2N/MoSe_2$ heterostructure, at different applied electric field (E_{field} = -0.5 and +0.5 V/Ang)*

Gibbs free energy

The $\Delta G_{\mathrm{H}*}$ is estimated by using the following equation (1) [1]:

$$\Delta G_{\mathrm{H}*} = \Delta E_{\mathrm{H}} + \Delta E_{ZPE} - T\Delta S_{\mathrm{H}} \quad (1)$$

where ΔE_{H} is the differential hydrogen adsorption energy, which is defined as:

$$\Delta E_{\mathrm{H}} = E(\mathrm{C_2N + H}) - E(\mathrm{C_2N}) - \mathrm{E(H_2)}/2 \quad (2)$$

where $E(\mathrm{C_2N + H}) - E(\mathrm{C_2N})$ are total energies of the C2N monolayer (or heterostructures) with and without hydrogen adsorption. T denotes room temperature and ΔS_{H} is considered as half of the entropy of H_2 in the gas phase under standard conditions. ΔE_{ZPE} denotes zero-point energy difference between adsorbed hydrogen and gas-phase hydrogen. $\Delta E_{ZPE} - T\Delta S_{\mathrm{H}}$ is about 0.24 eV.

[1] Shao, Y.; Shi, X.; Pan, H. Electronic, Magnetic, and Catalytic Properties of Thermodynamically Stable Two-Dimensional Transition-Metal Phosphides. *Chem. Mater.* **2017**, *29*, 8892–8900.
